# Life History Strategies Drive Meso‐Scale Distribution Patterns in Coastal Benthic Macroinvertebrates

**DOI:** 10.1002/ece3.70461

**Published:** 2024-10-25

**Authors:** Molline Natanah C. Gusha, Christopher D. McQuaid

**Affiliations:** ^1^ Department of Zoology and Entomology Rhodes University Makhanda (Grahamstown) South Africa; ^2^ Department of Ichthyology and Fisheries Science Rhodes University Makhanda (Grahamstown) South Africa; ^3^ South African Institute of Aquatic Biodiversity Makhanda (Grahamstown) South Africa

**Keywords:** biogeography, functional diversity, lifestyle, marine environment, reproduction, sea surface temperature, trade‐offs and spin‐offs, trait interactions

## Abstract

The environment shapes the spatial distribution of species, but species also comprise suites of traits which may indicate their adaptability to a specific environment. This forms the basis of trait biogeography studies. We thus examined how a species distribution is not only influenced by its environment and traits, but by interactions among its traits. Trait information was collected for 150 intertidal macroinvertebrates along a 3000 km environmental and biogeographic gradient on the South African coast. This information was analysed, as functional entities (FEs) were species performing similar functions that have the same trait values and were further condensed into two trait domains (Reproduction and Lifestyle). We then defined Life History Strategies (LHS) as specific combinations of Lifestyle and Reproduction FEs. Seven combinations of Lifestyle and Reproduction formed LHS that dominated total biomass. Some of these LHS were ubiquitous, while others showed geographic patterns across our west‐east environmental gradient. For Lifestyle, filter‐feeders exhibited high abundances on the East (subtropical, oligotrophic) and West (cool‐temperate, eutrophic) extremes of the biogeographic gradient, but differed between the two in size at reproductive maturity and larval development type. This similarity in functionality of feeding mechanism and mobility with different reproductive strategies suggests a trait trade–off (investment in one trait reduces resources for others) between the Reproduction and Lifestyle domains. Within the Reproduction domain, gonochoristic, annual planktotrophic reproduction was common across bioregions, reflecting spin‐offs (investment in one trait facilitates another trait) among these traits. Gonochoristic investment in less frequent episodic reproduction is another trade‐off, with investment in large size and delayed maturation being a trade–off for many reproductive cycles. Overall, although our data supports the habitat templet model (i.e., the importance of environmental drivers), it further indicates that species distribution patterns observed along the South African coast reflect strong trait interactions and biomass patterns related to their LHS.

## Graphical abstract

The environment shapes the spatial distribution of species, but species comprise suites of traits which may indicate their adaptability to the specific environment. We examined how the distribution of a species is influenced not only by its combination of traits, or Life History Strategy but also by interactions among its traits. Overall, although our data supports the habitat templet model (i.e., the importance of environmental drivers), it also indicates that species distribution patterns observed along the South African coast reflect strong trait interactions and biomass patterns related to their Life History Strategies.

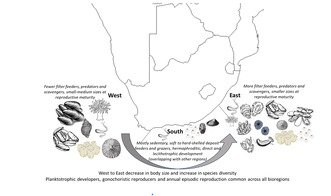

## 1 Introduction

Species composition is used to define biogeographic regions at meso (10 s–100 s km) and macro (100 s–1000 s km) spatial scales (Kreft and Jetz 2010). This facilitates our understanding of species distributions. However, defining the underlying drivers of these patterns within and between different biogeographical regions is not straightforward. This is because species responses to spatial or temporal variation in environmental conditions depend on many factors, including direct effects at the individual and population‐level, and indirect effects through changes in the distribution, abundance, and behaviour of competitors, predators, or conspecifics. The importance of integrating processes occurring at the level of individuals as well as those operating at the level of populations is well known (Giménez 2010), however, there are limitations to the methods used in these evaluations.

For example, the majority of studies of species assemblages across various spatial scales have primarily used taxonomic‐based indices to evaluate species presence, diversity, and richness (Tilman and Lehman 2001). Although these methods can accurately describe spatial and temporal differences in community composition and summarise information about the relative abundances of species within a community or a sample, they often ignore the differences among species in the traits that they exhibit and fail to capture the causal mechanisms underlying these patterns (Botta‐Dukát 2005). Thus, they cannot account for the many different ecological functions of the species that comprise communities and ultimately do not account for the implications of changes in biodiversity for the functioning of ecosystems and their services for humans (Hillebrand 2008).

An alternative approach is to use trait‐based methods that focus on the characteristics of species, rather than their identities. The premise of the trait‐based approach is that organism fitness is based on success in the fundamental life missions feeding, survival and reproduction and that the outcome of each of those missions depends on a few key traits (Brun, Payne, and Kiørboe 2016).

The trait‐based approach in biogeography or simply trait biogeography is well established particularly in plant ecology (e.g., Westoby et al. 2002) but its potential for animals has rarely been exploited. This may be because animal species comprise multiple traits which often exhibit a lot of plasticity, making characterising the traits in communities a time‐consuming and complex process. To circumvent this, one method of plying trait information at the community level has been through the use of functional groups (Mitwally 2022). Although general patterns of change in species diversity and distribution based on functional groups can be robust and predictable (e.g., Parmesan and Yohe 2003), predictions regarding the consequences of interactions among traits through this approach remain elusive.

Another promising approach to address this has been through the Life History Strategies (LHS) approach. The approach combines schedules of survival, development, and reproduction, shape how natural selection acts on species' heritable traits and organismal fitness (Stott et al. 2024). Life History Strategies have been defined as sets of co‐evolved traits which enable a species to deal with a range of ecological problems (see Stearns 1976). The terms ‘Life History Strategies’ and ‘suites of traits’ have been used interchangeably to emphasise the functional trait interlinkages within organisms (Verberk, Siepel, and Esselink 2008). Stearns focused on traits related to reproduction to define LHS. For the purposes of analysis herein, we focus on LHS rather than suites of traits but expand the term Life history strategy to include the Lifestyle trait domain as well as the Reproductive trait domain (see Siepel 1994; Verberk, Siepel, and Esselink 2008). Within the Reproductive trait domain, we consider four trait categories (reproductive type, frequency, size at reproductive maturity and three main larval development mechanisms: planktotrophic, direct and lecithotrophic development). For the Lifestyle trait domain, we examined five trait categories (body size, fragility or body form, feeding, mobility and preferred habitat position) as these directly affect the structure and dynamics of ecological networks and are related to ecosystem stability (Emmerson and Raffaelli 2004). From a regional perspective, the selection of these traits was informed by the robustness in species diversity of the rocky shore ecosystem of South Africa and we predicted that these traits would capture this diversity.

Further, critical to the LHS approach is also examining trade‐offs (investment in one trait leaves fewer resources for investment in others) and spin‐offs (investment in one trait increases the benefits or decreases the costs of investment in another). This concept was developed by Verberk, Siepel, and Esselink (2008) who worked on freshwater systems and its importance has been further stressed by Stott et al. (2024). We adapted this approach as it has the advantage of maximising the information provided by species by treating them as a suite of traits rather than as single traits or taxonomic units (Verberk, Siepel, and Esselink 2008). They suggested that the adaptive value of a trait depends on the other traits possessed by an organism. For example, there can be a trade‐off between body size and development time where with a constant supply of resources, allocating them to development shortens development time, while allocating resources to growth lengthens development time. The trade‐off is then between either small individuals with early maturation or large individuals with late maturation (Abrams et al. 1996). We therefore looked at the potential trade‐offs and spin‐offs among our nine trait categories.

From a biogeographic perspective, this understanding of LHS through their trade‐offs and spin‐offs is necessary for predicting large‐scale community responses to environmental change and how evolutionarily active biogeographic transition zones may respond (Beger et al. 2014). For example, species with different life histories may respond differently to gradients in environment conditions (Marko et al. 2010; Reynolds, Matthee, and Von der Heyden 2014; Hao et al. 2020). Therefore, to examine whether and how these LHS drive biogeographic patterns of intertidal species, we examined trait‐level responses of intertidal invertebrate communities along approximately 3000 km of the South African coast which is divided into three main bioregions.

Two major current systems, the Agulhas Current to the east and the Benguela Current to the west, dominate environmental conditions along this coast. The two create a distinct east‐west gradient of decreasing water temperature and increasing water column chlorophyll, reflected in three main biogeographic regions: the east, south and west coasts and the less studied transition zones between them, which we term the South‐West Overlap (SWO) and the South‐East Overlap (SEO) zones. Globally, shifts in climatic conditions are predicted to have significant effects on ecosystem processes through changes in species distributions. This reflects species tolerances and interactions and will affect the distribution of species traits, leading to cascading effects on ecosystem processes and services.

Given the known distributions of rocky shore species based on the oceanographic properties linked to the Agulhas and Benguela Currents on the east and west coasts, and the high endemism on the south coast, we hypothesised that there would also be a distinguishable difference in the spatial distribution of traits and how these traits interact within and among species, leading to different LHS Consequently, trait interactions would exhibit important spin‐offs and trade‐offs. In addition, the FEs framework also allows the computation of three indices (Functional Redundancy [FRed], Functional Overredundancy [FOver] and Functional Vulnerability [FVuln]) that can be used to identify the functional structure of communities. Here, we report the patterns of three indices that can be used to highlight and predict functional insurance within the ecosystem.

## 2 Materials and Methods

### 2.1 Study Area

The rocky intertidal ecosystem of South Africa exhibits well‐described alongshore gradients of abiotic conditions and biogeography. The coast comprises approximately 1300 km of rocky shores and 1700 km of sandy beaches (Bally, McQuaid, and Brown 1984). The coast is profoundly influenced by two major marine currents: the warm, oligotrophic southward flowing Agulhas current on the east and south coasts and the northward flowing Benguela on the west coast, which is characterised by frequent, strong wind‐driven coastal upwelling. This results in a clear west‐to‐east gradient of increasing sea temperatures and decreasing nutrient availability and levels of chlorophyll‐*a*, while rocky shore species tend to show Atlantic affinities to the west and Indian Ocean affinities to the east. Each of our sites was characterised by nearshore sea surface temperatures (SST) and chlorophyll‐a values derived from mean monthly values for satellite‐derived data over a 7‐year period as detailed elsewhere (Gusha and McQuaid *in review*). Although the specific details can differ among taxa (see Williams 1992; Turpie, Beckley, and Katua 2000; Bolton and Stegenga 2002), at the community level, the resulting patterns of species distributions allow the rocky coast to be broadly divided into West, East, and South bioregions with two transition zones, the SEO and the SWO, where the primary bioregions blend into one another.

**FIGURE 1:**
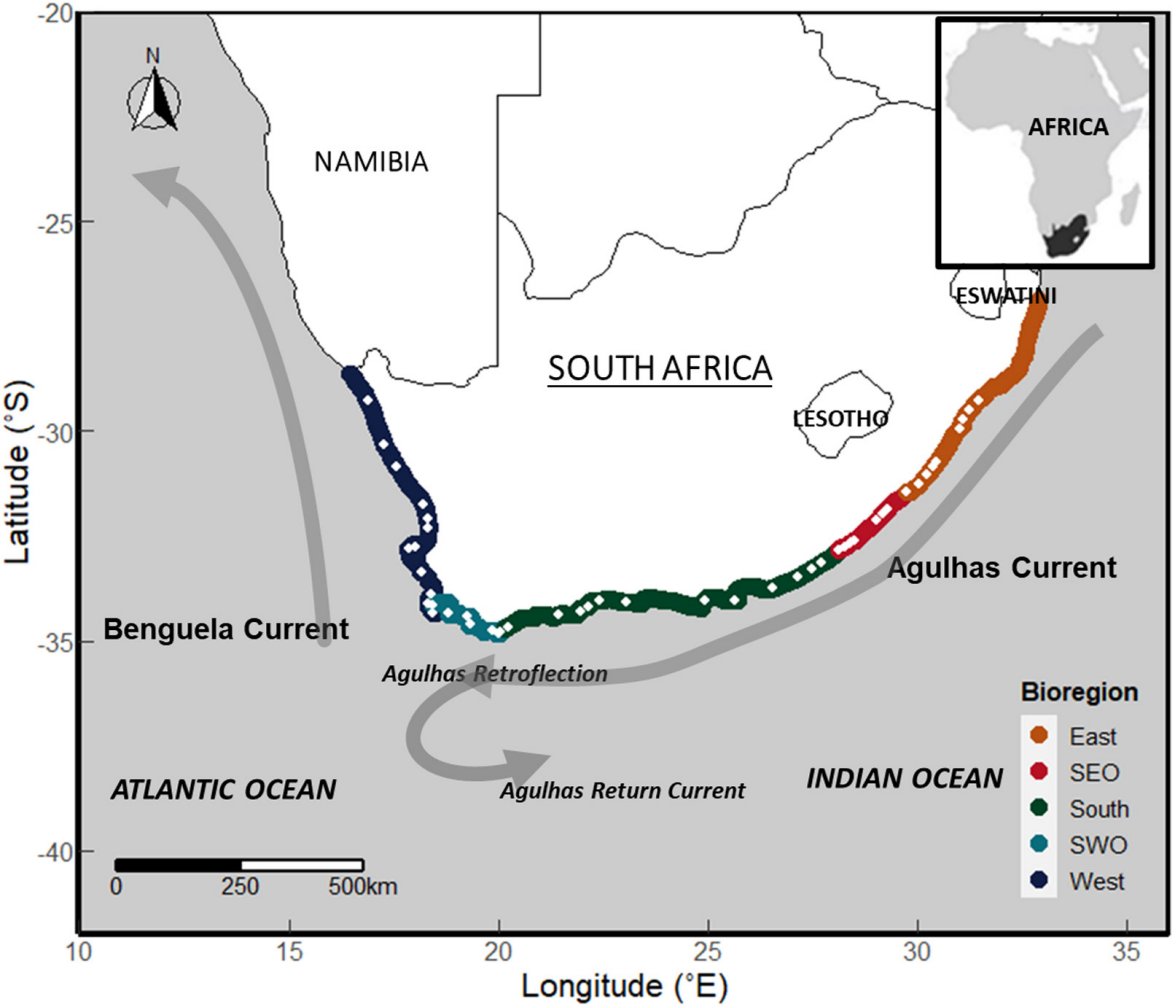
Map showing the 48 sites (represented by white dots) sampled along the coastline of South Africa. Sites were divided into 3 main bioregions and 2 transition zones (as indicated in the legend). Biodiversity along the coast is also significantly influenced by the physical properties of the two main currents the Agulhas on the east and the Benguela on the west which are in turn driven by the Indian and Atlantic Oceans, respectively.

Biomass data were collected from 48 wave‐exposed rocky shores sampled along the South African coastline, from Mabibi in the east to Port Nolloth in the west (Figure 1). Sites were roughly 50 km apart, with 8 sites on the east coast, 9 sites on the west coast, and 12 sites on the south coast. Within transition zones, sites were more closely spaced, with 8 in the south‐west (SWO) and 11 in the south‐east (SEO) overlap zones. Samples of 150 macroinvertebrates (> 10 mm) were collected during the summer months (October to December 2018).

### 2.2 Enumeration Weighting Methods, Field Sampling and Data Processing

Assessments of multivariate community structure of benthic assemblages may be based on either abundance or biomass data depending on the study objectives. Although abundance data are often used as they are less time consuming to collect, we based our analysis on biomass data as this is a more suitable metric for the assessment of an organism's influence on ecosystem functioning as it provides a better descriptor of the amount of carbon and other resources it represents (Bremner, Rogers, and Frid 2006). In addition, Saint‐Germain et al. (2007) highlight that biomass is a better indicator of the functionality of a species within a community and may provide a broader and more accurate picture of the processes driving changes in community structure, as it is strongly correlated with metabolism.

Sampling at each site was conducted between the sublittoral fringe and the upper balanoid zone or between the spring low tide and neap high tide levels (Branch et al. 2016), with tidal heights or zones classified following Branch and Branch (2018). All samples were collected during spring low tides, but, as some shores lacked certain zones, the number of zones sampled varied between three and four across sites. Quadrats (25 × 25 cm) were placed using a stratified random technique with transects to capture maximum diversity and were limited to flat or gently sloping surfaces. Tidal pools are effectively a separate ecosystem and thus were not included in the study. Before quadrats were sampled, they were photographed. At each site, four quadrats were collected from each of four transects approximately 200 m long, running parallel to the water line within each zone (Llanos et al. 2020). Specimens were collected using scrapers and chisels under the research permits (RES2018/16 and RES2019/30). For Dwesa and Mkambathi, which are within marine protected areas, we used the research permit (RA0269).

Due to the experimental design, we acknowledge that some of the highly mobile species such as amphipods were under‐sampled. Samples were preserved in 70% ethanol and later identified to the lowest taxonomic level possible (usually species), using taxonomic guides by Day 1974, monographs on polychaetes (Day 1967; Branch, 1974; Branch et al. 1991). Because specimens were preserved in ethanol, they were initially blotted with paper towels and allowed to air dry under a fume hood for 1–2 h before weighing. Species were counted and grouped into size classes by length or diameter (precision 0.1 mm) using digital callipers. Total wet weight (TWW) and shell‐free wet weight (SFWW) were measured. Dry shell‐free weight (DSFW) was obtained after drying to constant weight at 60°C for ~72 h. Ash‐free dry weight (AFDW) was obtained following incineration in a muffle furnace (500°C, ~6–8 h). Average individual TWW, SFWW, DSFW and AFDW for each size class were obtained by dividing the total weight by the number of individuals in each size class. All statistical analyses were based on AFDW biomass, considered the most ecologically meaningful measure of biomass (Crisp 1984). The decision to measure actual AFDW follows the work by Ricciardi and Bourget (1998) who suggested estimating AFDW on fresh samples rather than conversion factors whenever possible.

### 2.3 Trait Selection and the Fuzzy Coding Technique

We quantified the LHS of our species by assembling trait data into an ascending three‐tier hierarchy of modality, category, and domain (Table 1, MarLIN 2006; Costello et al. 2015). The trait information of all collected species was captured using the fuzzy coding technique (Chevene, Doleadec, and Chessel 1994). Although some studies extend the scale from 0 to 5, the fuzzy coding approach of 0 to 3 is the original approach and is preferred for marine systems where 0 means a complete lack of affinity with a particular trait, 1 indicates low affinity, and 2 a higher affinity with similar or partial affinity for another. A score of 3 indicates exclusive affinity to a particular modality (Bremner, Rogers, and Frid 2003; Vinagre et al. 2019). The fuzzy coding technique compensates for the different confidence levels of trait information obtained from the literature (Chevene, Doleadec, and Chessel 1994). Because most intertidal species exhibit high level of plasticity, the method also aims to address challenges associated with the direct assignment of a taxon to a single trait attribute which may not fully capture the species trait information (Mondy and Usseglio‐Polatera 2014).

**TABLE 1:** Trait domains (in italics), categories (in bold), and modalities used in this study.

*Lifestyle*	Trait code	*Reproduction*	Trait code
**Feeding group**		**Reproductive frequency**	
Filter‐feeders	t1.1	Annual protracted	t6.1
Grazers/herbivores	t1.2	Annual episodic	t6.2
Deposit feeders	t1.3	Continuous	t6.3
Carnivores/predators/omnivores	t1.4	Semelparous	t6.4
Scavengers	t1.5		
**Adult size**		**Reproductive type**	
Extra small (< 10 mm)	t2.1	Spores/asexual	t7.1
Small (10–30 mm)	t2.2	Gonochoristic	t7.2
Medium (30–50 mm)	t2.3	Sequential hermaphrodites	t7.3
Large (50–70 mm)	t2.4	Simultaneous hermaphrodites	t7.4
Extra large > 70 mm	t2.5		
**Mobility**		**Size at reproductive maturity**	
Sessile	t3.1	Extra small (< 10 mm)	t8.1
Sedentary	t3.2	Small (10–30 mm)	t8.2
Crawler	t3.3	Medium (30–50 mm)	t8.3
Swimmer	t3.4	Large (50–70 mm)	t8.4
Burrower	t3.5	Extra large > 70 mm	t8.5
**Preferred habitat position**		**Larval development mechanism**	
Infratidal	t4.1	Direct developer	t9.1
Low	t4.2	Planktotrophic	t9.2
Mid	t4.3	Lecithotrophic	t9.3
High	t4.4		
Pools	t4.5		
**Fragility/body form**			
Soft/exposed/brittle	t5.1		
Intermediate	t5.2		
Robust	t5.3		

It is important to note that because age information is limited for most of the species sampled and recognising that most marine invertebrates reproduce within the first 1–2 years (Gosselin and Qian 1997), we used the ‘size at reproductive maturity’ as a proxy for age at reproductive maturity. The size classes obtained from the Biological Traits Information Catalogue were adapted based on expert advice (Branch and Branch, 2018) to account for rocky shore macroinvertebrates. Although marine benthic invertebrates exhibit various types of larval development, for simplicity we focused on the main three which include (l) development through pelagic larval stages of varying durations (2) development within the parental organism; (3) development up to juvenile bottom‐dwelling stage inside the egg‐capsules spawned by the parents into external bottom environments. Importantly, since the trait information was captured using the fuzzy‐coding approach, the selection of trait interrelations that were then grouped into LHS was based on trait modalities with complete affinity, that is, a score of 3.

### 2.4 Life History Strategies Selection

We evaluated the distribution of the most unique functional traits of invertebrate biomass using the Functional Entities (FEs) framework (Magneville et al. 2022). Under this framework, traits can be grouped into entities of unique combinations. We then used these FEs to compose possible LHS potentially driving the meso‐scale distribution of intertidal macroinvertebrates and identified any critical trade‐offs and spin‐offs among them.

### 2.5 Data Analysis

**FIGURE 2:**
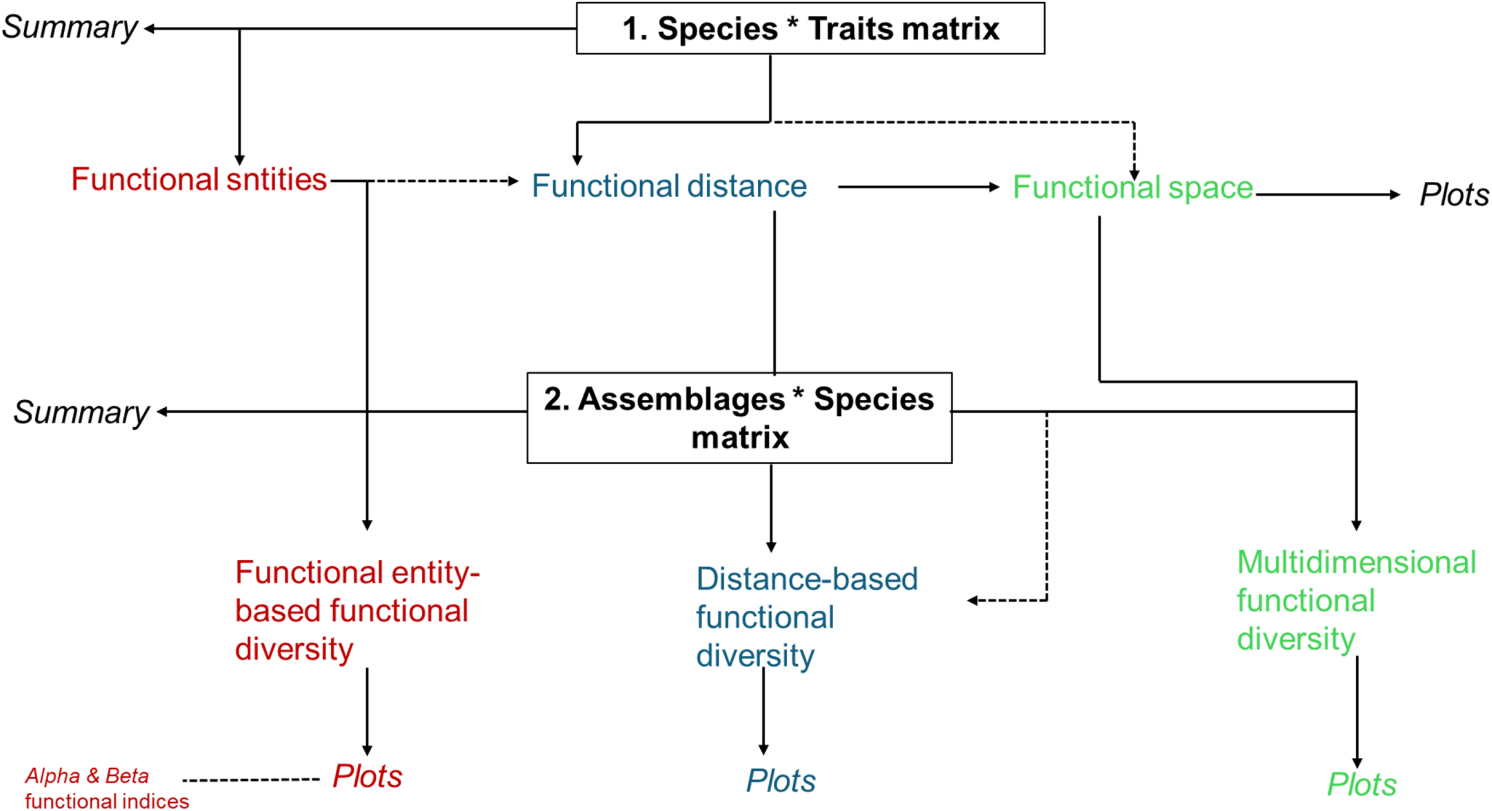
The three colour coded (red, blue and green) frameworks that can be used for estimating functional diversity indices adapted from the mFD package by Magneville et al. (2022). Each framework uses two datasets (a species by traits matrix and a assemblages by species matrix). Our study is based on the red framework.

Using the mFD package, we first estimated the FEs within the rocky shore species assemblage using one of three possible frameworks; the FEs based on functional diversity indices (Magneville et al. 2022). This framework groups species or FEs into one framework, the other two frameworks not considered in this study are the pairwise trait‐based distances between species and the species coordinates in a multidimensional space framework (see Figure 2). Importantly, given our objective of identifying potential LHS, the other two frameworks were beyond the scope of this work. The functional entities (hereafter FEs) are a way of grouping species with unique combinations of functional traits when species are described with categorical, ordinal traits or fuzzy coded traits. Mouillot et al. (2014), highlighted that when only categorical and ordinal traits are used to describe species, there is a finite number of combinations of trait values making it likely that some species from the pool will share the same trait values and can therefore be clustered into FEs.

Functional entities were computed for each bioregion and each domain (see Figures S1 and S2 and Tables S2 and S3). We then used the FE trait combinations to identify and construct LHS (see Tables 1 and 2). The FEs also calculate three indices (FRed, FOver and FVuln). FRed which is an index that reflects the average number of species per FE. FOver reflects the proportion of species in excess in species‐rich FEs, that is, it represents the percentage of species that fill FEs above the mean level of FRed. FVuln reflects the proportion of FEs with only one species. We used these indices to evaluate the vulnerability of these ecosystems. All analyses were performed in R version 4.3.0 (R Core Team 2022).

**TABLE 2:** A and B represent Lifestyle and Reproduction FEs, respectively. C highlights the 12 Life History Strategies.

**A. Lifestyle traits: All FEs include small size**
1. Hard shell sedentary filter feeders
2. Hard shell sedentary grazers
3. Soft‐intermediate sedentary grazers
4. Small to extra‐large, sedentary, filter feeders, predators, and scavengers
**B. Reproduction traits: All FEs include annual episodic reproduction and small size at maturity**
A. Gonochoristic, planktotrophic
B. Hermaphroditic, direct and lecithotrophic
C. Small to medium at maturity, gonochoristic, hermaphroditic, planktotrophic
**C. Life History Strategy combinations**
1/A: Hard shell, sessile filter‐feeders, small at maturity, gonochoristic, planktotrophic
1/B: Hard shell, sessile filter‐feeders, small at maturity, hermaphroditic, direct and lecithotrophic
1/C: Hard shell, sessile filter‐feeders, small‐medium at maturity, gonochoristic, hermaphroditic, planktotrophic
2/A: Hard shell sedentary grazers, gonochoristic, planktotrophic
2/B: Hard shell sedentary grazers, hermaphroditic, direct and lecithotrophic
2/C: Hard shell sedentary grazers, small to medium at maturity, gonochoristic, hermaphroditic, planktotrophic
3/A: Soft‐intermediate sedentary deposit feeding, gonochoristic, planktotrophic
3/B: Soft‐intermediate sedentary grazers, hermaphroditic, direct and lecithotrophic
3/C: Soft‐intermediate sedentary grazers, small to medium at maturity, gonochoristic, hermaphroditic, planktotrophic
4/A: Medium to extra‐large, hard shell sedentary grazers, filter feeders, predators, and scavengers, gonochoristic, planktotrophic
4/B: Medium to extra‐large, sedentary hard shell, grazers, filter feeders, predators, and scavengers, hermaphroditic, direct and lecithotrophic
4/C: Medium to extra‐large, sedentary, filter feeders, predators, and scavengers, small to medium at maturity, gonochoristic, hermaphroditic, planktotrophic

*Note:* For both Lifestyle and Reproduction domains, all categories share common features with an extra category (e.g., number 4 for Lifestyle, number C for Reproduction) that includes additional features.

## 3 Results

### 3.1 Spatial Patterns of Macroinvertebrate Biomass, and Traits

Table S1 gives the species exhibiting the highest (top 10) and lowest (bottom 5) biomass values across all sites and within each bioregion. Apart from two zoanthid species (*Zoanthus durbanensis* and *Z. natalensis*), which are restricted to the eastern parts of the coastline, the native mussel *Perna perna*, the native barnacle *Octomeris angulosa* and the invasive mussel *Mytilus galloprovincialis* generally had the highest abundances. Of the species showing the lowest values for biomass, none were found in multiple bioregions.

### 3.2 Functional Entities Across Bioregions

We identified a total of 147 FEs based on unique combinations of nine categorical functional traits to classify the 150 identified rocky shore species. The Reproduction trait domain had three while the Lifestyle domain had four FEs that included more than one species. We therefore report on the patterns of these seven FEs.

#### 3.2.1 Lifestyle Trait Domain

For Lifestyle traits, all bioregions showed few (0–3) FEs containing more than one species, with the great majority of FEs containing only one species (indicated in red in Figure S2). As a result, redundancy (FRed) and vulnerability (FVuln) were close to one in all cases. Similarly, because only two to three FEs in each region contained more than one species, the range of functional over redundancy (FORed), indices was low (0–0.048), except for the South coast where FORed was 0.027. In all cases, the FEs with > 1 species were combinations of 7 FEs (FE1–FE7). For the East coast they were FE1, FE2 and FE4 (FORed = 0.048), for the southeast overlap (SEO) FE2, FE3 and FE4 (FORed = 0.045) and for the South coast they were FE3 and FE5 (FORed = 0.027). In the southwest overlap (SWO) region no FEs contained > 1 species so FRed and FVuln were 1, while FORed was 0. For the West coast, FE5, FE6 and FE7 contained > 1 species (FORed = 0.042).

For reproduction traits (Figure S2), there was a marked west–east increase, from 4 to 10, in the number of FEs with > 1 species. The western sites (i.e., SWO and West, Figure S2d,e) had the fewest such FEs (four and five, respectively), the South coast had 8 (Figure S2c), while the SEO and East coasts each had 10 (Figures S2a,b). Each bioregion had ~2 LHS identified from the FEs (see Table S3).

As a result, FRed was notably higher, for Reproduction traits (1.153–1.22) than Lifestyle traits (1.029–1.053, cf. Figure S1). FORed values were also much higher across all bioregions (0.851–0.932) than for Lifestyle traits (0.027–0.048). FVuln showed an East–West increase from 0.851 to 0.932. Overall, Lifestyle traits showed higher FVuln (0.954–0.971) than Reproduction traits (0.851–0.932). For Lifestyle traits, the south coast showed the highest FVuln while for the Reproduction traits, the west coast had the highest FVuln.

## 4 Discussion

Using pooled functional traits in a life history approach highlights the potential of condensing species trait information to understand underlying mechanisms when the large‐scale nature of a study is logistically challenging. We found a plethora of FEs (reflecting the diversity of organisms involved), but the vast majority were represented by only 1 species, with only 4 Lifestyle and 3 Reproduction FEs being represented by > 1 species. Together, these seven FEs combine to offer 12 potential LHSs of which five did not contribute significantly to biomass. A few of the seven LHS dominating total biomass were found throughout the coast, while others were more restricted across this biogeographic/environmental gradient.

Life History Strategies 1A, 1C, 2B and 3C were ubiquitous across bioregions and dominated biomass. 2A showed a western eutrophic affinity (SWO and West), while 3A showed an eastern oligotrophic affinity (SEO and East). Lastly, 4C was present in all bioregions except the South coast, suggesting an effect of latitude. The results also indicated that Reproduction traits showed higher redundancy levels than Lifestyle traits, with the two domains exhibiting maximum FVuln (and minimum FRed) in different bioregions. In addition to the alongshore biogeographic gradient in environmental conditions, the vertical transition from marine to terrestrial conditions represents a second, particularly steep gradient. This too influenced the predominance of different LHS.

### 4.1 Ubiquitous Across All Bioregions

#### 4.1.1 LHS 1A. Hard‐Shell, Sedentary Filter‐Feeders, Small‐Medium Body Size at Sexual Maturity, Gonochoristic, Planktotrophic Larval Development

This Life History Strategy was one of the most ubiquitous across all bioregions. The species having these trait combinations and possessing the highest biomass were mussels (*Perna perna, Mytilus galloproviancilis, Choromytilus meridionalis*) and rock oysters (*Saccostrea cucullata*). The combination of limited mobility (sedentary) and a hard shell/protective layer is especially important for filter feeding intertidal species in exposed rocky shores. In isolation, filter feeding is an optimised feeding mechanism minimising energy costs (Riisgård and Larsen 2010). Therefore, when coupled with small size at reproductive maturity or early reproduction, this ensures population persistence in areas where resources might be limiting and become a bottleneck to attaining bigger sizes at reproduction.

These species have planktotrophic development and although recruitment can be unpredictable, this mode of reproduction provides a critical spin‐off for sessile species where prone to high predation as saturation recruitment can swamp predators (Sams and Keough 2007). The combination of filter‐feeding with planktotrophic development thus seems to be adaptive across a wide range of environmental conditions with little influence of trait interactions. Paine and Levin (1981) also highlighted that, in communities dominated by filter‐feeders, predators and environmental instability may play a vital role in preventing monopolisation of the habitat by a single dominant species. This may be true in our study considering that these traits were found across bioregions offering different environmental constraints.

#### 4.1.2 LHS 1C. Hard‐Shell, Sessile Filter‐Feeders, Small at Maturity, Gonochoristic, Hermaphroditic, Planktotrophic Larval Development

This trait combination was also ubiquitous and is similar to LHS 1A but differs in that these species may be either gonochoristic or hermaphroditic. Species exhibiting the highest biomass possessing these trait combinations were two native barnacles (*Tetraclita serrata* and *Octomeris angulosa*). These barnacle species are often separated along two gradients of environmental conditions. Across the vertical, within‐shore gradient, species with similar functional traits often replace each other along the sharp low‐ to high‐shore gradient of stress and these barnacles are generally separated by shore height. They both also showed shifts in body size across this gradient, with larger individuals lower in the shore within their respective zones. The two are also separated across gradients of wave exposure, with *O. angulosa* being more tolerant of wave action both within and among shores (Boland 1997). Other macrospatial scale studies on barnacles have attributed such differences to reproductive periodicity, availability of larvae, nearshore current patterns and coastal topography (Gaines, Brown, and Roughgarden 1985). On a local scale, recruitment of barnacles has been related to predation during the planktonic larval phase (Roughgarden and Gaines 1987), the abundance of adults, substratum heterogeneity (Chabot and Bourget 1988), and biological interactions resulting in high post‐settlement mortality.

#### 4.1.3 LHS 2B. Hard‐Shell Sedentary Grazers, Hermaphroditic, Direct or Lecithotrophic Larval Development

This trait combination was also common across all bioregions with the biomass of such species being dominated by siphonariid limpets especially *Siphonaria capensis*. Although overlapping across all regions, species exhibiting this LHS showed specific biogeographic affiliations, with some found to the East and others to the West. Simultaneous hermaphroditism in siphonariids and protandrous hermaphroditism in some of the patellid limpets (e.g., Le Quesne and Hawkins 2006) is a critical spin–off rather than a trade–off to having gonochoristic reproduction as it is linked to providing a selective advantage under environmentally unsuitable habitats. By functioning as the sex with the higher fecundity in a particular age bracket, an organism can increase its reproductive potential relative to lifetime males or females (Warner 1975).

Direct developing species often have low offspring counts but higher offspring survival and such development is a trade–off between high *per capita* investment in offspring and low fecundity. Winemiller and Rose (1992) classify species with this trade‐off as equilibrium strategists of various sizes from small to large in body size, low fecundity per spawning event, but high juvenile survivorship largely due to high parental care and small clutch size. Coupled with their hard shells which offer significant protection relative to exposed/soft‐shelled species, this strategy allows species to increase in abundance and potentially dominate biomass. Lecithotrophy has similar trade‐offs to direct development. This is because when food/energy is low or unpredictable, reproductive success is maximised by allocating energy into fewer, larger offspring.

#### 4.1.4 LHS 3C. Soft‐Intermediate Sedentary Deposit Feeding, Gonochoristic, Planktotrophic

LHS 3C was also found across all bioregions. The most common taxa with this strategy were the tube‐dwelling polychaetes (*Gunnarea* sp.), which are among the most abundant marine metazoans in benthic environments. These polychaetes are mostly gonochoristic with hermaphroditism usually secondary within some families. Like LHS 1A, the combination of gonochorism, annual episodic reproduction and planktotrophic development also seems to be adaptive across a wide range of environmental conditions with little influence of trait interactions. This is corroborated by Giangrande (1997), who highlighted that there is a strong relationship between environmental cues such as temperature and photoperiod and reproduction success among polychaetes.

### 4.2 Longitudinal Patterns

#### 4.2.1 LHS 2A. Hard‐Shell Sedentary Grazers, Gonochoristic, Planktotrophic

The biomass for species with this LHS was dominated by large patellid limpets (e.g., *Cymbula granatina* and *Scutellastra argenville*) and these are especially abundant (often at enormous densities) on the west coast. The existence of high chlorophyll levels on the west coast is linked to the high nutrient levels induced by upwelling. In parallel with high phytoplankton levels, the west‐east decrease in nutrient levels results in a similarly clear gradient in the productivity of intertidal epilithic algae. This in turn is reflected in the biomass and individual sizes of limpet grazers (Bustamante et al. 1995).

#### 4.2.2 LHS 3A. Soft‐Intermediate Sedentary Grazers, Small to Medium at Maturity, Gonochoristic, Hermaphroditic, Planktotrophic

The sea cucumber (*Roweia frauenfeldii*) exhibits LHS 3A and was predominantly found on the eastern parts of the coast. Echinoderms are typically gonochoric and broadcast their gametes in the water column, where fertilisation occurs. In many cases, the gametogenic cycle and/or spawning activity of echinoderms appears to be broadly correlated with environmental factors. For example, spawning has been found to occur during the rainy season when chlorophyll‐*a* concentrations were maximal (Leite‐Castro et al. 2016). Other important environmental factors in holothuroids that have been determined to act independently or in combination at various stages of the reproductive cycle include temperature, photoperiod (Hamel, Himmelman, and Dufresne 1993), the lunar cycle (Mercier, Ycaza, and Hamel 2007), phytoplankton blooms (Wigham et al. 2003) and diffusible chemical signals (Hamel and Mercier 1996). As annual episodic reproducing species, the dependence of reproductive timing on environmental cues is also linked with pre‐spawning aggregative behaviour as a strategy to help synchronise gametogenesis, with phytoplankton blooms acting as a trigger for gamete release optimising larval feeding.

### 4.3 Latitudinal Patterns

#### 4.3.1 LHS 4C. Medium to Extra‐Large, Sedentary, Filter‐Feeders, Predators and Scavengers, Small to Medium at Maturity, Gonochoristic, Hermaphroditic, Planktotrophic

Species exhibiting high biomass and LHS 4C belonged to a variety of taxa. These include zoanthids (*Zoanthus* spp. and *Palythoa* spp.), the predatory whelks (*Burnupena* spp.), and sand anemones (*Bunodactis reynaudi*). These species were found at similar latitudinal ranges at both ends of our biogeographic gradient, that is, across all bioregions except the South coast. This LHS included a particularly wide range of feeding types (including filter‐feeding, deposit‐feeding, scavenging and carnivory), with different types of species dominating the biomass of this LHS in different places. The biomass of this LHS was dominated by predatory whelks and anemones on the west coast, and by zoanthids to the east. The entire latitudinal range of our study area is small (6°–7° of latitude), and it is unclear why this LHS was not represented on the south coast.

### 4.4 Inter‐and Intra‐Trait Interactions and Meso‐Scale Intertidal Species Distribution

In this study, we considered two trait domains: Lifestyle and Reproduction. Although for the purposes of analysis, we considered the two as separate, they can influence each other through trait interactions. For example, feeding type can affect the viability of different modes of reproduction. Dominance or frequency of occurrence of a feeding modality is influenced by the quantity and nature of food availability and possible interspecific competition for food or space. Hines (1992) suggested that the distribution of certain reproductive traits may be constrained by factors influencing lifestyle traits. In our study, both planktotrophy and size at reproductive maturity broadly decreased from West to East across the eutrophic/oligotrophic biogeographic gradient. In contrast, no lifestyles showed a West–East gradient, suggesting that reproduction is more responsive to abiotic conditions. One plausible reason for the former is that the sites selected were all relatively wave exposed and a plethora of work including McQuaid and Branch (1984, 1985), McQuaid and Branch (1985) and Bustamante et al. (1995) have shown that intertidal species with lifestyle traits such as filter feeding often the highest diversity along the entire coastline. Considering intertidal species diversities on these shores have not changed notably (Branch et al. 2016, 2018; Branch and Branch 2018), this pattern may be the similar our study, leading to no major changes in lifestyle trait distribution along the coastline.

Overall, we interpret the inter‐trait biogeographic pattern in these two aspects of Reproduction and Lifestyle as reflecting two key elements. First, a West–East decrease in food availability is expected to result in slower growth and a consequent need to reach sexual maturity at a smaller size, or risk not reaching maturity at all. For example, the primary determinant of growth rate in mussels is the availability is food (Seed and Richardson 1990), and growth rates of mussels are positively correlated with phytoplankton availability at multiple scales. This in turn reflects both chlorophyll levels in the water (Steffani and Branch 2003; Xavier, Branch, and Wieters 2007) and the rate of supply, which is affected by wave exposure or current velocity (Archambault, McKindsey, and Bourget 1999; Blanchette, Broitman, and Gaines 2006), shore height (Connor and Robles 2015) and even by centimetre scale variation in hydrodynamics (McQuaid and Mostert 2010). Second, none of these species are mobile across more than minimal spatial scales. This can be compensated for through enhanced ability to compete for space, for example through asexual reproduction. Pineda et al. (2012) suggested that, where adult sessile species are unable to escape unfavourable conditions, the success of early stages is critical for local population persistence. For species with dispersive larvae, population success doesn't depend on adult feeding type (as adults can survive periodically low food availability), but on the availability of phytoplanktonic food and its effect on larvae. Spawning of planktotrophic larvae can be triggered by chlorophyll levels (Abe, Sato‐Okoshi, and Endo 2011) and low food availability can lead to reduced larval growth, decreased larval survival (Wolfe et al. 2015) and reduced larval supply (Lara et al. 2016). Even if larvae survive to settlement, poor condition following exposure to low food availability, especially early on (Phillips 2004), can result in poor performance following settlement, including increased juvenile mortality (Phillips 2002). Larval supply is in turn linked to population structure and even adult longevity (McQuaid and Phillips 2006). For both early maturity and reliance on planktotrophic larvae, the effect of the environment on reproduction is an indirect one arguably mediated through the effect of the environment on traits such a feeding type.

In addition to the horizontal gradient, another key habitat property that influenced the distribution of the LHS observed was vertical zonation across the intense environmental gradient offered by rocky shores. For instance, the various filter feeders recorded in this study were found at different shore heights but with varying trait combinations such as soft bodied species on the low shore and those with robust body forms that can tolerate longer periods of desiccation on the mid to higher shore heights. As previously shown by Awad, Griffiths, and Turpie (2002), we also observed that for body size, larger filter feeders (mussels, large barnacles) occurred lower on the shore with smaller filter feeders (small barnacles) higher up. Overall, the species showing the greatest biomass across our environmental gradient were functionally similar in terms of feeding mechanism and mobility, but differed in terms of reproductive strategy, indicating an inter‐trait domain trade–off between reproduction and lifestyle strategies.

Another important intra‐trait interaction within the reproductive domain concerned small body sizes, annual episodic reproductive frequency and gonochoristic and/or hermaphroditic reproduction. This combination was common in all three of the main bioregions and seems to reflect trade‐offs among these traits. We suggest that gonochoristic investment in less frequent episodic reproduction is a trade‐off among size, development, and delayed maturation, because investment in size and maturation is a trade–off for many reproductive cycles. Hermaphroditism was also found in species across most bioregions, with most of species being of small body sizes, suggesting another inter‐trait domain trade‐off. This is because the ability to generate sufficient gametes or mates (in the case of hermaphroditism) to maximise the probability of reproductive success requires a minimum body size and a minimum maturation period (Young 2003).

## 5 Conclusion

Using a trait‐based approach to understanding species distributions allows us to separate the effects of function and biogeography; the function of a species is determined by its combination of traits, while its identity reflects its biogeographic affinity or origins. Nevertheless, evaluating trait patterns along large spatial scales is not straightforward as each species comprises a suite of interlinked traits with some being more responsive to variability in the physical and biological environment than others. Some combinations of traits, or LHS, were ubiquitous across our biogeographic/environmental gradient, while others were more restricted. Although, we found evidence to support the habitat templet model by Townsend and Hildrew, 1994 (i.e., the importance of the nature of the habitat), our results show that species biomass across the South African coastline were also strongly influenced by trait interrelations. Overall, the life history strategies of benthic macroinvertebrates were shaped by the physical environment, but also reflected trait interactions both within and between trait domains.

## Author Contributions

**Molline Natanah C. Gusha:** conceptualization (equal), data curation (lead), formal analysis (lead), writing – original draft (lead), writing – review and editing (equal). **Christopher D. McQuaid:** conceptualization (equal), funding acquisition (lead), supervision (lead), validation (equal), writing – review and editing (equal).

## Conflicts of Interest

The authors declare no conflicts of interest.

## Supporting information


Appendix S1

## Data Availability

The data that support the findings of this study are openly available in ResearchGate at https://doi.org/10.13140/RG.2.2.17079.09120.
