# Supplementary material for: Life History Strategies Drive Meso‐Scale Distribution Patterns in Coastal Benthic Macroinvertebrates
Source: Ecol Evol. 2024 Oct 25;14(10):e70461. doi: 10.1002/ece3.70461 (PMC11502938; doi:10.1002/ece3.70461)
Supplement: Supplementary file 1 — Appendix S1 [file ECE3-14-e70461-s001.docx]

**Ecology and Evolution**

**Research Article**

**Supplemental Information**

**Life history strategies drive meso-scale distribution patterns in coastal benthic macroinvertebrates**

**Molline Natanah. C. Gusha*^1,2.3^, Christopher D. McQuaid^1^**

**Functional entity patterns across bioregions**

**Life style trait domain**

**a)**

**^
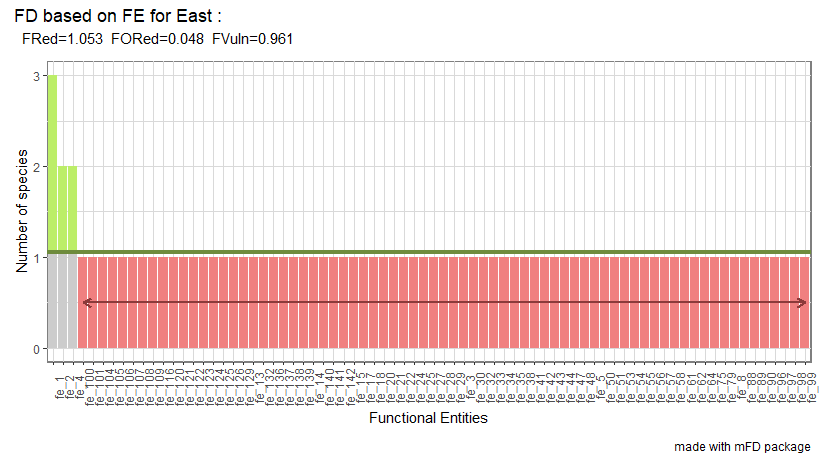
^**

**b)**

**^
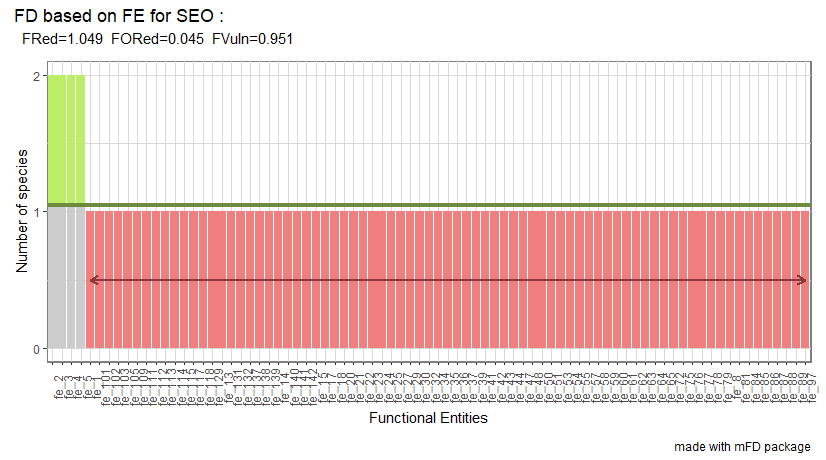
^**

**c)**

**^
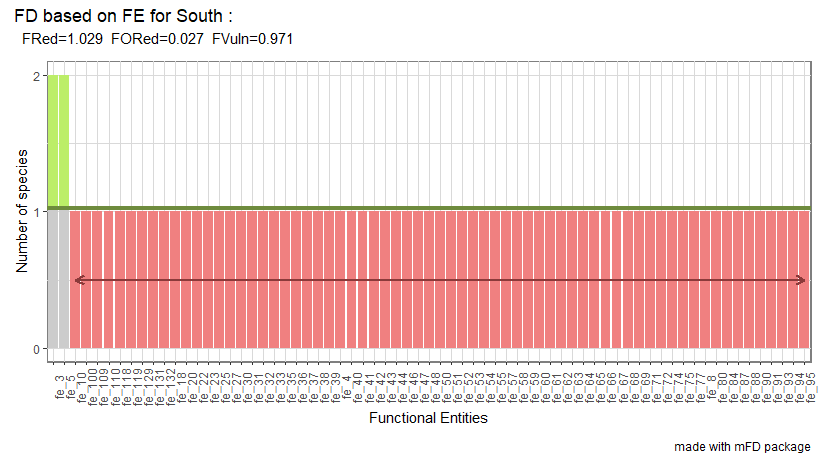
^**

**d)**

**^
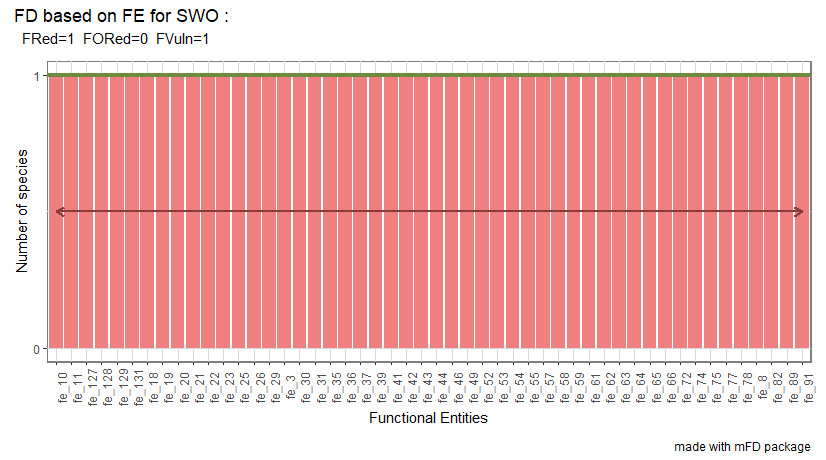
^**

**e)**

**^
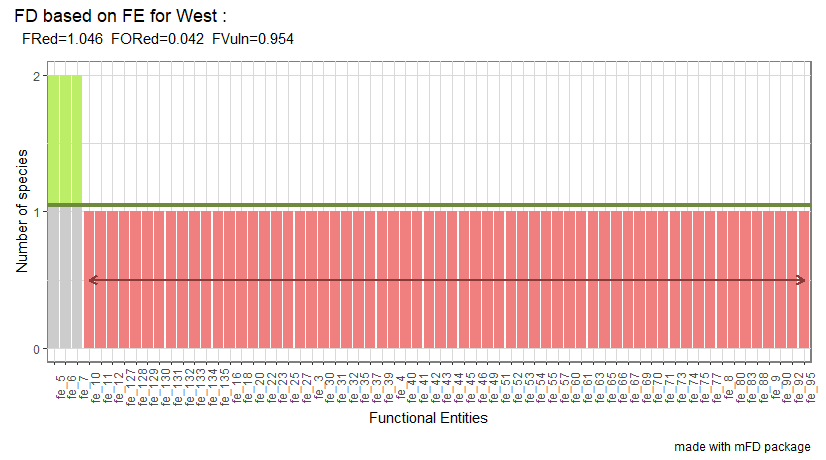
^**

Figure S1. Functional Entities calculated across each bioregion. The trait combinations are listed in Table S2. The x-axis shows the variety and total number of FEs present within each bioregion. The y axis shows the number of species within each computed FE. The grey and green bars highlight the FEs with more than one species while the red highlights FEs with only one species.

**Reproduction trait domain**

**a)**

**^
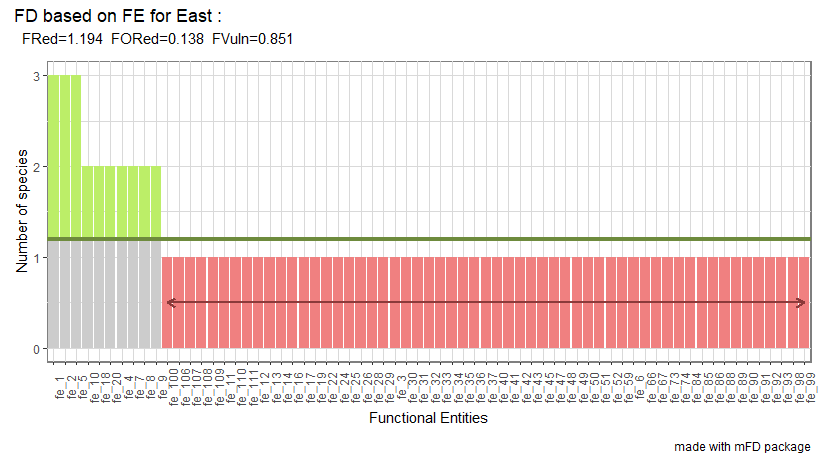
^**

**b)**

**^
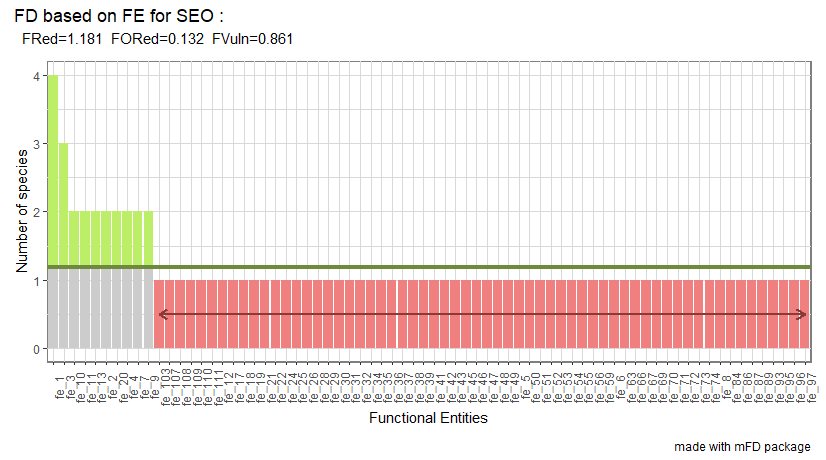
^**

**c)**

**^
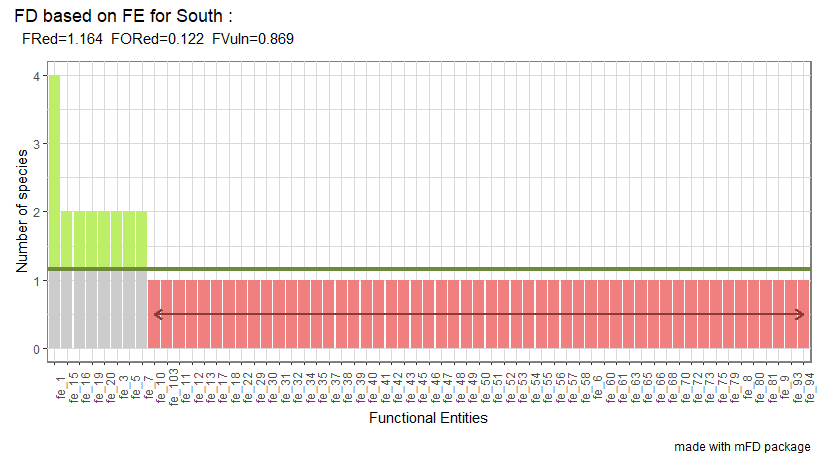
^**

**d)**

**^
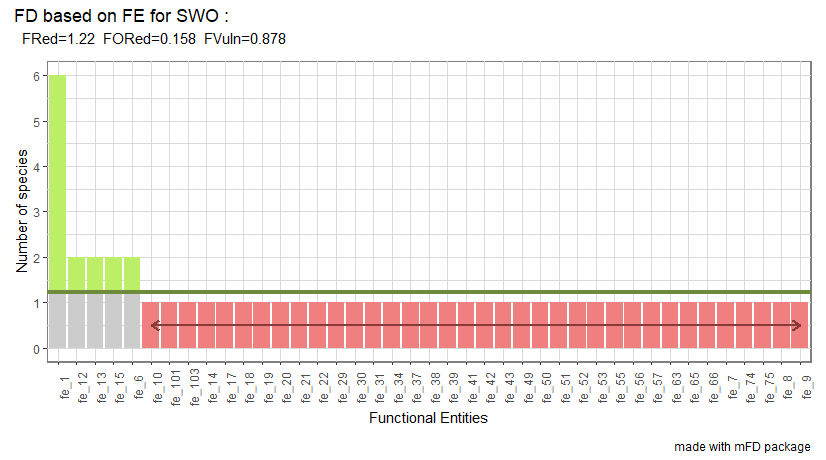
^**

**e)**

**^
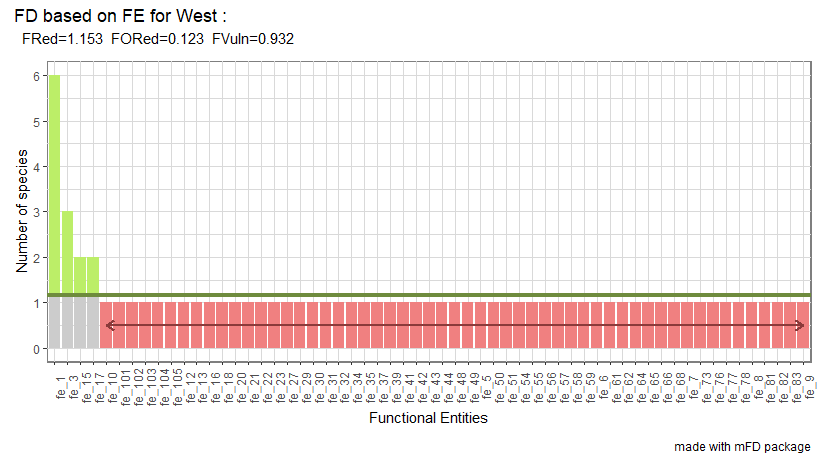
^**

Figure S2. Functional Entities calculated across each bioregion. The trait combinations are listed in Table S3. The x-axis shows the variety and total number of FEs present within each bioregion. The y axis shows the number of species within each computed FE. The grey and green bars highlight the FEs with more than one species while the red highlights FEs with only one species.

| **Highest biomass** | **All sites** | **East** | **South-East** | **South** | **South-West** | **West** |
| --- | --- | --- | --- | --- | --- | --- |
|  | *Perna perna* | *Perna perna* | *Zoanthus natalensis* | *Chthamalus dentatus* | *Bunodactis reynaudi* | *Mytilus galloprovincialis* |
|  | *Mytilus galloprovincialis* | *Zoanthus natalensis* | *Perna perna* | *Perna perna* | *Mytilus galloprovincialis* | *Choromytilus meridionalis* |
|  | *Zoanthus natalensis* | *Palythoa natalensis* | *Octomeris angulosa* | *Mytilus galloprovincialis* | *Scutellastra cochlear* | *Cymbula granatina* |
|  | *Choromytilus meridionalis* | *Octomeris angulosa* | *Saccostrea cuccullata* | *Tetraclita serrata* | *Choromytilus meridionalis* | *Bunodactis reynaudi* |
|  | *Octomeris angulosa* | *Saccostrea cuccullata* | *Scutellastra cochlear* | *Octomeris angulosa* | *Octomeris angulosa* | *Scutellastra cochlear* |
|  | *Tetraclita serrata* | *Zoanthus durbanensis* | *Siphonaria serrata* | *Gunnarea gaimardi* | *Scutellastra granularis* | *Scutellastra argenvillei* |
|  | *Scutellastra granularis* | *Roweia frauenfeldii* | *Argobuccinum pustulosum* | *Scutellastra granularis* | *Cymbula oculus* | *Aulacomya atra* |
|  | *Palythoa natalensis* | *Scutellastra natalensis* | *Roweia frauenfeldii* | *Sedentaria sp* | *Aulacomya atra* | *Octomeris angulosa* |
|  | *Scutellastra cochlear* | *Cellana capensis* | *Cellana capensis* | *Siphonaria serrata* | *Gunnarea gaimardi* | *Burnupena lagenaria* |
|  | *Cymbula granatina* | *Gunnarea gaimardi* | *Gunnarea gaimardi* | *Siphonaria capensis* | *Siphonaria capensis* | *Gunnarea gaimardi* |
| **Least biomass** |  |  |  |  |  |  |
|  | *Tylothais savignyi* | *Eurythoe complanata* | *Scutellastra obtecta* | *Grapsus tenuicrustatus* | *Rathbunixa occidentalis* | *Nucella squamosa* |
|  | *Diodora crucifera* | *Ischnochiton oniscus* | *Nucella dubia* | *Rhyssoplax polita* | *Afrocominella capensis simoniana* | *Vaughtia fenestrata* |
|  | *Neorhynchoplax bovis* | *Afropinnotheres dofleini* | *Ischnochiton textilis* | *Parisocladus perforatus* | *Spirobranchus kraussi* | *Afropinnotheres dofleini* |
|  | *Grapsus tenuicrustatus* | *Neorhynchoplax bovis* | *Scutellastra barbara* | *Urothoe grimaldii* | *Urothoe grimaldii* | *Tetraclita serrata* |
|  | *Afrolittorina Africana* | *Sipunculida sp* | *Glycera tridactyla* | *Charybdis sp* | *Ischyromene huttoni* | *Diodora crucifera* |

**Table S1** **Highest and least species recorded across bioregions according to biomass.**

Table S2: List of Functional entities computed for lifestyle traits using the mFD package. The Life History Strategy column represents most common traits identified within each bioregion.

| **Lifestyle traits** | | | |
| --- | --- | --- | --- |
| **Bioregion** | **FE code** | **Functional entity** | **Life History Strategy** |
|  |  |  |  |
| East | FE1 | Filter feeders, Adult size-Small, Sessile, Low shore, Soft/Exposed/Brittle body form, | Sessile, filter feeders, small adult size, fringe & low zones, soft, intermediate and robust body form |
|  | FE2 | Filter feeders, Adult size -Small, Sessile, Low shore, Intermediate body form |  |
|  | FE4 | Grazers & herbivores, Adult size -Small, Sessile, Robust body form. |  |
| SEO | FE2 | Filter feeders, Adult size -Small, Sessile, Low shore, Intermediate body form; | Sessile, filter feeders, small adult size, fringe & low zones, soft, intermediate and robust body form |
|  | FE3 | Grazers & herbivores, Adult size -Small, Sessile, Low shore, Robust body form |  |
|  | FE5 | Grazers & herbivores, Adult size -Extra-Small, Crawlers, Pools, Soft/Exposed/Brittle body form. |  |
|  | FE4 | Grazers & herbivores, Adult size -Small, Sessile, Robust body form |  |
| South | FE3 | Grazers & herbivores, Adult size -Small, Sessile, Low shore, Robust body form | Sedentary grazers and herbivores, small adult size, pool habitat, robust body form |
|  | FE5 | Grazers & herbivores, Adult size -Extra-Small, Crawlers, Pools, Soft/Exposed/Brittle body form. |  |
| SWO |  | No FEs |  |
| West | FE5 | Grazers & herbivores, Adult size -Extra-small, Crawlers, Pools, Soft/Exposed/Brittle body form | Sedentary grazers and herbivores, scavengers, filter feeders, small and large adult size, pool habitat, robust body form |
|  | FE6 | Scavengers, Adult size -Extra-Small, Crawlers, Pools, Soft/Exposed/Brittle body form |  |
|  | FE7 | Filter feeders, Adult size -Extra-large, Sessile, Low shore, Robust body form |  |

Table S3: List of Functional entities computed for reproduction traits using the mFD package. The Life History Strategy column represents most common traits identified along each bioregion.

| **Reproduction traits** | | | |
| --- | --- | --- | --- |
| **Bioregion** | **FE code** | **Functional entity** | **Life History Strategy** |
| **East** | FE1 | Annual episodic, Gonochoric, Planktotrophic, Small size at reproductive maturity | Small size at reproductive maturity, annual episodic, gonochoristic, and planktotrophic development |
|  | FE2 | Continuous, Asexual, Direct developers | Asexual, direct developers and continuous reproducers |
|  | FE5 | Annual episodic, Continuous, Gonochoric, Planktotrophic |  |
|  | FE18 | Annual episodic, Gonochoric, Planktotrophic, Small size at reproductive maturity |  |
|  | FE20 | Annual episodic, Gonochoric, Planktotrophic, Extra small size at reproductive maturity |  |
|  | FE4 | Continuous, Gonochoric, Planktotrophic, Extra small size at reproductive maturity |  |
|  | FE7 | Annual episodic, Sequential hermaphroditism, Planktotrophic, Medium size at reproductive maturity |  |
|  | FE8 | Annual episodic, Gonochoric, Planktotrophic, Small size at reproductive maturity |  |
|  | FE9 | Continuous, Planktotrophic, Small size at reproductive maturity |  |
| **SEO** | FE1 | Annual episodic, Gonochoric, Planktotrophic, Small size at reproductive maturity | Small size at reproductive maturity, hermaphroditism, annual episodic, direct and lecithotrophic development, |
|  | FE3 | Continuous, Direct developers, Extra small size at reproductive maturity | Extra small size at reproductive maturity, gonochoric, continuous reproducers, planktotrophic developers, |
|  | FE5 | Annual episodic, Continuous, Gonochoric, Planktotrophic |  |
|  | FE10 | Annual episodic, Sequential hermaphroditism, Planktotrophic |  |
|  | FE11 | Sequential hermaphroditism, Permanent hermaphroditism, Small size at reproductive maturity |  |
|  | FE15 | Annual episodic, Gonochoric, Direct developer, Small size at reproductive maturity |  |
|  | FE16 | Annual episodic, Gonochoric, Lecithotrophic, Small size at reproductive maturity |  |
|  | FE18 | Annual episodic, Gonochoric, Planktotrophic, Medium size at reproductive maturity |  |
|  | FE20 | Annual episodic, Gonochoric, Planktotrophic, Extra small size at reproductive maturity |  |
|  | FE4 | Continuous, Gonochoric, Planktotrophic, Extra small size at reproductive maturity |  |
|  | FE8 | Annual episodic, Gonochoric, Planktotrophic, Small size at reproductive maturity |  |
| **South** | FE1 | Annual episodic, Gonochoric, Planktotrophic, Small size at reproductive maturity | Small size at reproductive maturity, annual episodic, continuous reproduction, and lecithotrophic larval development |
|  | FE5 | Annual episodic, Continuous, Gonochoric, Planktotrophic | Extra small size at reproductive maturity direct developers, continuous reproduction. |
|  | FE16 | Annual episodic, Gonochoric, Lecithotrophic, Small size at reproductive maturity |  |
|  | FE19 | Continuous, Gonochoric, Planktotrophic, Small size at reproductive maturity |  |
|  | FE20 | Annual episodic, Gonochoric, Planktotrophic, Extra small size at reproductive maturity |  |
|  | FE3 | Continuous, Direct developers, Extra small size at reproductive maturity |  |
|  | FE7 | Annual episodic, Sequential hermaphroditism, Planktotrophic, Medium size at reproductive maturity |  |
| **SWO** | FE1 | Annual episodic, Gonochoric, Planktotrophic, Small size at reproductive maturity | Small-medium size at reproductive maturity, annual episodic, gonochoristic, planktotrophic continuous reproductive frequency |
|  | FE12 | Annual protracted, Gonochoric, Planktotrophic, Small size at reproductive maturity |  |
|  | FE13 | Annual episodic, Permanent hermaphrodites, Planktotrophic, Small size at reproductive maturity |  |
|  | FE15 | Annual episodic, Gonochoric, Direct developer, Small size at reproductive maturity |  |
|  | FE6 | Annual episodic, Gonochoric, Direct developers, Extra-small size at reproductive maturity |  |
| **West** | FE1 | Annual episodic, Gonochoric, Planktotrophic, Small size at reproductive maturity | Similar to SWO |
|  | FE3 | Continuous, Direct developers, Extra small size at reproductive maturity |  |
|  | FE15 | Annual episodic, Gonochoric, Direct developer, Small size at reproductive maturity |  |
|  | FE17 | Annual episodic, Gonochoric, Planktotrophic, Medium size at reproductive maturity |  |
